# Mating-type switching by homology-directed recombinational repair: a matter of choice

**DOI:** 10.1007/s00294-018-0900-2

**Published:** 2018-10-31

**Authors:** Geneviève Thon, Takahisa Maki, James E. Haber, Hiroshi Iwasaki

**Affiliations:** 1 Department of Biology, BioCenter, University of Copenhagen, Copenhagen, Denmark; 2 Institute of Innovative Research, Tokyo Institute of Technology, Tokyo, Japan; 3 Department of Biology and Rosenstiel Basic Medical Sciences Research Center, Brandeis University, Waltham, MA 02453 USA; 4 Department of Life Science and Technology, School of Life Science and Technology, Tokyo Institute of Technology, Tokyo, Japan

**Keywords:** Chromatin structure, Gene conversion, Histone modifications, Homology-directed repair, Mating-type switching, Recombination

## Abstract

In eukaryotes, all DNA transactions happen in the context of chromatin that often takes part in regulatory mechanisms. In particular, chromatin structure can regulate exchanges of DNA occurring through homologous recombination. Few systems have provided as detailed a view on this phenomenon as mating-type switching in yeast. Mating-type switching entails the choice of a template for the gene conversions of the expressed mating-type locus. In the fission yeast *Schizosaccharomyces pombe*, correct template choice requires two competing small recombination enhancers, *SRE2* and *SRE3*, that function in the context of heterochromatin. These two enhancers act with the Swi2/Swi5 recombination accessory complex to initiate strand exchange in a cell-type-specific manner, from *SRE2* in M cells and *SRE3* in P cells. New research indicates that the Set1C complex, responsible for H3K4 methylation, and the Brl2 ubiquitin ligase, that catalyzes H2BK119 ubiquitylation, participate in the cell-type-specific selection of *SRE2* or *SRE3*. Here, we review these findings, compare donor preference in *S. pombe* to the distantly related budding yeast *Saccharomyces cerevisiae*, and contrast the positive effects of heterochromatin on the donor selection process with other situations, where heterochromatin represses recombination.

## Introduction

**Fig. 1:**
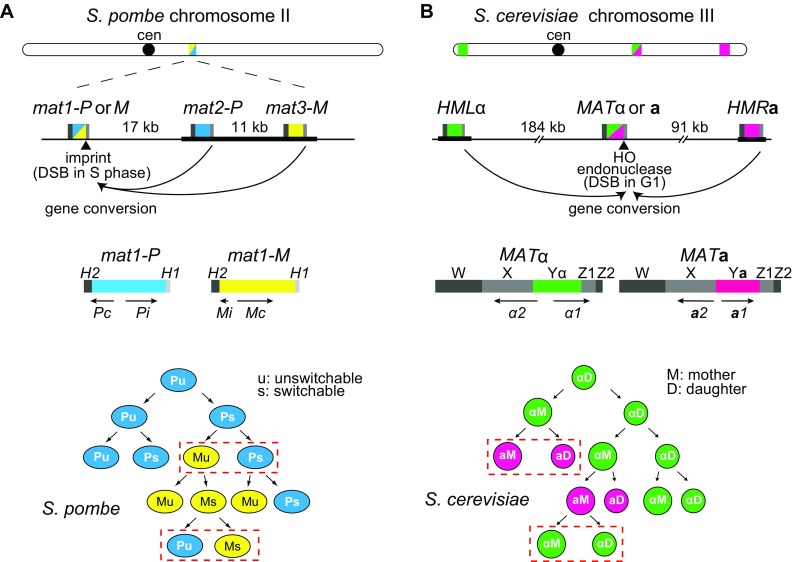
Mating-type cassettes and mating-type switching patterns in *S. pombe* (**a**) and *S. cerevisiae* (**b**). In *S. pombe*, the expressed *mat1* locus switches between *mat1-P* and *mat1-M* due to gene conversions by the linked *mat2-P* and *mat3-M* silent loci. In *S. cerevisiae*, the expressed *MAT* locus switches between *MAT*α and *MAT***a** due to conversions by *HML*α and *HMR***a**. Silent heterochromatic regions are indicated by thick black lines. Gene conversions are initiated in both cases by a DNA break at the expressed locus followed by strand invasion at one of the silent cassettes. Strand invasion occurs at homology boxes present at the three cassettes: *H1* in *S. pombe* and *Z1* in *S. cerevisiae*. In *S. pombe*, the DNA break is formed during DNA replication in cells that have acquired an imprint during the previous DNA replication (switchable cells), and only one of the newly synthesized chromatids undergoes conversion producing one switched and one unswitched progeny (examples framed in red in cell pedigree). In *S. cerevisiae*, the DNA break is formed and used in the G1 phase of the cell cycle, exclusively in mother cells due to restricted expression of the HO endonuclease in mother cells, this produces pairs of switched cells after DNA replication and cell division (examples framed in red). The silent cassettes that contain mating-type information opposite to the expressed cell type are preferentially chosen in both yeasts, giving rise to the observed high efficiency of switching in the progeny of switching-competent cells; however, donor preference is not determined by the mating-type-specific sequences in the donors

The fission yeast *S. pombe* and the budding yeast *S. cerevisiae* both switch mating type during vegetative growth (Fig. 1). *Schizosaccharomyces pombe* switches between the P and M cell types by replacing the content of the expressed *mat1* cassette with information copied from one of the two linked silent cassettes, *mat2-P* or *mat3-M* (reviewed by Klar et al. 2014). *Saccharomyces cerevisiae* switches between the **a** and α cell types by replacing the content of the *MAT* locus located on chromosome III with information copied from the silent *HML*α or *HMR***a** donors located at either end of the same chromosome (reviewed by Lee and Haber 2015). In both yeasts, mating-type switching is initiated by a double-strand break (DSB), which is repaired by ectopic homologous recombination (gene conversion); but the origin of the DSB is quite different in the two distantly related yeasts. In fission yeast, a single-strand nick or gap is “imprinted” at *mat1* in the S phase of the cell cycle, so that the following round of DNA replication creates a DSB on one of the two sister chromatids (Arcangioli and de Lahondes 2000; Dalgaard and Klar 2001). In budding yeast, a site-specific HO endonuclease creates the break at *MAT* (Kostriken et al. 1983; Strathern et al. 1982), normally at the end of the G1 phase (Nasmyth 1983). In both yeasts, mating-type switching depends on the key homologous recombination protein Rad51 and on its principal mediator Rad52 (Aboussekhra et al. 1992; Malone and Esposito 1980; Ostermann et al. 1993; Roseaulin et al. 2008). Briefly, a Rad51-coated end of the DSB promotes strand invasion into a short region of homology shared by the *mat1*/*MAT* locus and its donors (*H1* in *S. pombe*, Z in *S. cerevisiae*), followed by copying of the mating-type-specific P/M or Y**a**/Yα regions and terminating in the *H2* or W regions of homology shared on the opposite side of the DSB (Fig. 1).

The precise molecular mechanisms of switching differ greatly between the two yeasts, but they have in common their remarkable efficiency, and the fact that in both cases heterochromatic templates are used as donors for the gene conversions of the expressed cassette. Thus, while observations in many organisms have indicated that recombination can be inhibited in heterochromatic regions to limit exchanges between repetitive elements, the mating-type systems provide a more nuanced view of the effects of chromatin structure on recombination by showing how various histone modifications, in particular heterochromatic modifications in fission yeast, can instead promote the choice of gene conversion donors and efficient recombination.

## Chromatin factors required for directional mating-type switching in fission yeast

Three genetic screens have produced *S. pombe* mutants with donor choice defects (Egel et al. 1984; Thon et al. 2005; Maki et al. 2018). Donor choice, or directionality of mating-type switching, refers to a selection process, whereby the *mat2-P* cassette is preferentially chosen in M cells to convert the expressed *mat1* locus, while the *mat3-M* cassette is preferentially chosen in P cells, resulting in equal proportions of the two cell types (Fig. 1) (Egel and Eie 1987; Klar 1990; Miyata and Miyata 1981). Heterochromatin defects are at the source of impaired donor choice for many mutants. Thus, a mutant in the chromodomain protein Swi6, homolog of the major heterochromatin protein HP1 in other organisms, was obtained in the first genetic screen for mating-type switching deficient mutants (Egel et al. 1984) and subsequently characterized for its directionality defects (Jakociunas et al. 2013; Jia et al. 2004; Thon and Klar 1993). Another strategy identified the multi-subunit Clr4-containing complex (CLRC) responsible for the methylation of histone H3K9 as necessary for proper donor choice, by searching for mutations that reduce heterologous switching in the wild-type *h*^*90*^ strain but increase heterologous switching in the *h*^*09*^ strain, where the *mat2* and *mat3* cassette contents are swapped (Thon et al. 2005). More recently, we used a sensitive high-throughput fluorescence microscopy approach to find mutants with aberrant ratios of P and M cells, among which directionality mutants were identified (Maki et al. 2018). Table 1 presents the mutants obtained in this screen that have defects in histone-modifying enzymes. The list emphasizes that accurate donor selection requires the H3K9 modification pathway and various histone deacetylases (HDACs), as well as modifications whose relevance to switching had previously not been suspected. In particular, the screen identified the Set1C complex responsible for histone H3K4 methylation and the ubiquitin ligase Brl2 responsible for histone H2BK119 ubiquitylation. Mutations in these factors lead to less extreme biases in mating-type distributions than mutations in heterochromatin factors.

**Table 1:** Histone modifiers required for the directionality of mating-type switching in *S. pombe*

Modification	Name	Description	Complex
HDAC	Sir2	Sirtuin family (NAD-dependent) histone deacetylase Sir2	
	Clr1	SHREC complex subunit Clr1	SHREC
	Clr2	Chromatin silencing protein Clr2	
	Clr3	Histone deacetylase (class II) Clr3	
H3K9 methyl transferase	Clr4	Histone H3 lysine methyltransferase Clr4	CLRC
	Rik1	CLRC ubiquitin ligase complex WD repeat protein Rik1	
	Raf1	CLRC ubiquitin ligase complex WD repeat subunit Raf1	
	Raf2	CLRC ubiquitin ligase complex subunit Raf2	
H3K4 methyl transferase	Set1	Histone lysine methyltransferase Set1	Set1C
	Swd1	Set1C complex subunit Swd1	
	Swd2	Set1C complex subunit Swd2.1	
	Swd3	WD repeat protein Swd3	
	Ash2	Ash2-trithorax family protein	
	Spf1	Set1C PHD Finger protein Spf1	
H2BK119 ubiquitination	Brl2	Ubiquitin-protein ligase E3 Brl2	HULC

The factors listed were identified, among other factors, in a search for directionality mutants (Maki et al. 2018)

**Fig. 2:**
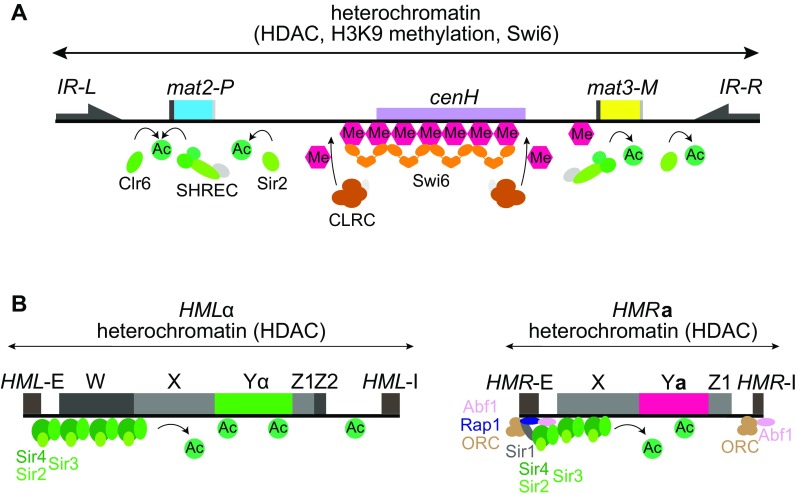
Heterochromatin at the donor loci. **a** In *S. pombe*, histone deacetylation by several HDACs (SHREC, Clr6, Sir2) together with histone H3K9 methylation by CLRC forms heterochromatin over a 20 kb domain between the *IR-L* and *IR-R* boundaries. Following histone modification, the chromodomain protein Swi6 associates with the entire 20 kb region. *cenH* is an RNA-interference heterochromatin nucleation center with centromere homology. **b** In *S. cerevisiae*, heterochromatin results from histone deacetylation and association of Sir proteins in two smaller domains, each < 3 kb, at the *HML* and *HMR* loci. At each cassette, the E and I silencers recruit various combinations of DNA-binding proteins, ORC, Rap1, and Abf1, to initiate heterochromatin formation

Heterochromatin normally occupies ~ 20 kb in the mating-type region including the *mat2-P* and *mat3-M* cassettes (Fig. 2). It is formed by the histone H3K9 methyltransferase CLRC and by several HDACs including the NAD-dependent Sir2, the Class I HDAC Clr6, and the SHREC complex containing the Class II HDAC Clr3. Heterochromatinization culminates in the recruitment of two HP1 homologs, Swi6 and the chromodomain protein Chp2. An important function of heterochromatin is to silence *mat2-P* and *mat3-M*, but due to redundant mechanisms of silencing, a strong repression remains in CLRC, Swi6, and Chp2 mutants, while a small derepression is seen in SHREC mutants (Hansen et al. 2011; Maki et al. 2018; Thon and Verhein-Hansen 2000). A derepression in the same order occurs in *swi6Δ* and *chp2Δ* mutants, yet *chp2Δ* mutants do not display switching defects (Thon and Verhein-Hansen 2000), showing that directionality defects in heterochromatin mutants are not a mere consequence of deregulated cell-type expression. Rather, heterochromatin regulates the use of two competing recombination enhancers, *SRE2* and *SRE3* (see below).

A novel aspect revealed by our recent screen is that the directionality of mating-type switching also relies on Set1C and its partner, the Brl2 ubiquitin ligase (Maki et al. 2018). Individual deletions of six of the eight Set1C subunits alter donor choice (Table 1). The identified mutations severely impair methylation of histone H3K4, unlike mutations in the two subunits that were not identified in the screen, Shg1 and Sdc1 (Mikheyeva et al. 2014). Furthermore, a necessary step for H3K4 methylation is ubiquitylation of histone H2BK119; both modifications fail in the Brl2 mutant (Zofall and Grewal 2007). Thus, collectively, the obtained mutants points to histone H3K4 as a methylation substrate relevant to directionality and indicate that Set1C and Brl2 might contribute together with heterochromatin to the regulation of *SRE2* and *SRE3*.

## The cell-type-specific recombination enhancers *SRE2* and *SRE3*

**Fig. 3:**
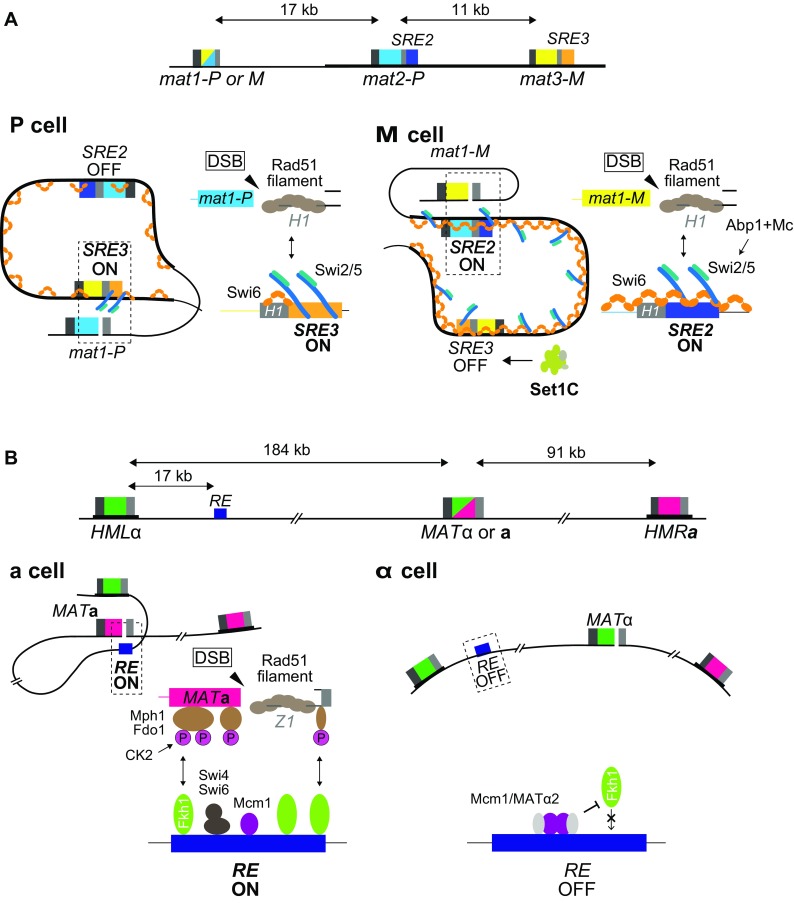
Choice of recombination enhancers in *S. pombe* and *S. cerevisiae*. **a***Schizosaccharomyces pombe SRE2* and *SRE3* enhancers. In P cells, the Swi2/Swi5 complex is exclusively associated with the recombination enhancer *SRE3*; this facilitates Rad51-mediated strand invasion at the *H1* region of *mat3* and thus promotes switching from *mat1-P* to *mat1-M*. In M cells, the Swi2/Swi5 complex is more broadly associated with the entire heterochromatic domain, this association requires the chromodomain protein Swi6. Under these conditions, the *SRE2* enhancer is preferred over *SRE3* leading to switching from *mat1-M* to *mat1-P*. The use of *SRE3* in M cells might be limited by Set1C. **b***Saccharomyces cerevisiae RE* enhancer. In *MAT***a** cells, *RE*, bound by multiple copies of the Fkh1 protein, establishes a physical contact with the *MAT* locus through the interaction of Fkh1’s phospho-threonine binding domain with phosphorylated proteins located at or near the DSB; these proteins include Mph1 and Fdo1. RE binding at the site of the DSB shortens the distance between *MAT***a** and *HML*α, favoring Rad51-mediated recombination between *MAT* and *HML*α. In *MAT*α cells, binding of Fkh1 to *RE* is disrupted by the repressor, Mcm1/Matα2; consequently, *HMR***a**, closer to *MAT*, is then preferred

The small Swi2- dependent recombination enhancers, *SRE2* and *SRE3*, are necessary for the cell-type-specific choice of *mat2-P* in M cells and *mat3-M* in P cells (Jakociunas et al. 2013; Jia et al. 2004) (Fig. 3). The molecular mechanism of action and regulation of these elements are not precisely known. During DNA replication, a single-ended double-strand break formed at the *mat1* locus by the incoming fork initiates a template switch for leading-strand synthesis (Arcangioli and de Lahondes 2000; Dalgaard and Klar 2001). The broken molecule invades one of the silent cassettes at a 59 bp region of sequence homology, the *H1* box. *SRE2* and *SRE3* are adjacent to the *H1* boxes at *mat2-P* and *mat3-M*, respectively; each element is < 500 bp. In all likelihood, *SRE2* and *SRE3* direct donor choice by favoring strand invasion at their adjacent *H1* box. Deletion of one enhancer results in the near exclusive use of the other cassette, whereas substitution of one enhancer with the other results in relatively efficient heterologous switching, indicating that each enhancer can stimulate recombination effectively in both P and M cells and that the two enhancers compete with each other when both are present (Jakociunas et al. 2013). Their relative use would be biased by differences in chromatin structure and differential association of the switching factor Swi2 with the mating-type region in the two cell types to achieve directionality.

## Chromatin-driven association of recombination factors with *SRE2* and *SRE3*

The preponderance of evidence indicates that Swi2 has a role in strand exchange, similar to the related Sfr1 protein (Swi five-dependent recombination repair protein 1) for which a role in Rad51-mediated strand exchange has been extensively documented (Akamatsu et al. 2003, 2007; Argunhan et al. 2017; Haruta, et al. 2006; Ito et al. 2018; Kurokawa et al. 2008). Swi2 and Sfr1 each engage the recombination accessory factor Swi5 to form respectively the Swi2/Swi5 and Sfr1/Swi5 complexes that physically and functionally interact with the Rad51 recombinase. Another key physical interaction of Swi2, with strong implications for mating-type switching, is with the chromodomain protein Swi6 (Akamatsu et al. 2003; Jia et al. 2004). Through this interaction, Swi2 bridges heterochromatin to the recombination apparatus (Fig. 3). Sfr1 on the other hand fails to interact with Swi6 and has no obvious role in mating-type switching (Akamatsu et al. 2003), while Rad51 is essential for the repair of the break at *mat1* and thus the viability of switching-competent cells (Roseaulin et al. 2008).

A role for the Swi2/Swi5 complex in directionality is indicated by the requirement for both factors for efficient mating-type switching (Egel et al. 1984) and by the differential association of the complex with the mating-type region in P and M cells, in a manner that precedes break formation at *mat1* (Fig. 3) (Jia et al. 2004). In P cells, the complex is predominantly at *SRE3*, perhaps through direct binding of Swi2 to *SRE3* DNA via the Swi2 AT-hook motifs. The complex shows a broader distribution in M cells, where it covers the whole *mat2*-*mat3* region including both *SRE2* and *SRE3*. Unlike for its localized association with *SRE3*, the extended association of Swi2/Swi5 in M cells requires Swi6 (Jia et al. 2004). During mating-type switching, interaction between the Rad51 presynaptic filament originating from *mat1* with Swi2/Swi5 bound to one of the *SRE* elements would stimulate the strand-exchange activity of the recombinase.

How differential Swi2 recruitment or activity might occur and lead to differential use of *SRE2* and *SRE3* in the two cell types and how other factors might influence use of *SRE2* and *SRE3* remain open questions. It has been proposed that two Swi2 isoforms exist, a shorter form of the protein would be produced exclusively in M cells following transcription initiation of the *swi2* gene at an internal promoter under control of the Abp1 protein and the *mat1-M* product Mc, and this smaller form would be necessary for the broad distribution of Swi2 over the mating-type region in M cells (Yu et al. 2012) (Fig. 3). The two Swi2 isoforms have not been detected at the protein level (Jia et al. 2004; Matsuda et al. 2011), but further investigations might reveal them. Other differences between P and M cells are that the Swi2 and Swi5 proteins are in greater abundance in M cells (Matsuda et al. 2011) and more Swi6 is detected at the mating-type region in M cells (Noma et al. 2001), both of which might contribute to the increased and broader association of Swi2 and Swi5 observed in M cells and to the differential use of *SRE2* and *SRE3* in the two cell types.

## Possible points of action for Brl2 and Set1C

A central question relates to the selective use of the *SRE2* recombination enhancer in M cells, where the Swi2/Swi5 complex is detected at both *SRE2* and *SRE3*. Greater proximity of the *mat2* donor to *mat1* in the linear DNA was first suggested as a determinant factor (Jia et al. 2004), yet linear proximity might not be crucial, because *mat2* is still preferred when *mat2* and its *SRE2* enhancer are moved away from *mat1* by swapping the *mat2* and *mat3* donor cassettes together with their respective enhancers (Jakociunas et al. 2013). Directionality still operates in these cells. One possibility is that the chromatin structure specific to M cells allows *SRE2* to outcompete *SRE3*. Our new study (Maki et al. 2018) indicates that Set1C and Brl2 might be part of this mechanism by inhibiting *SRE3*. Cells whose *SRE2* element is deleted use *SRE3*, leading to a strong bias towards M cells in the cell population (Jakociunas et al. 2013). This bias is even more pronounced in the *set1Δ* and *brl2Δ* mutants indicating Set1 and Brl2 normally limit recombination at *SRE3* (Maki et al. 2018). At another locus, the *ste11* gene promoter, H2BK119 ubiquitylation and H3K4 methylation prevent chromatin remodeling by the RSC complex (Materne et al. 2015, Materne et al. 2016), suggesting remodeling might also modulate recombination in the mating-type region.

Other effects are not excluded. As for the Abp1 protein, that both binds the mating-type region (Aguilar-Arnal et al. 2008) and regulates Swi2 transcription (Yu et al. 2012), Set1C and Brl2 might both act directly in the mating-type region and indirectly by controlling the production of Swi2 or other factors. In the mating-type region, some effects might be due to Set1C and Brl2 modulating Clr4-dependent methylation of histone H3K9.

## A different mechanism for the directionality of mating-type switching in *S. cerevisiae*

The picture that emerges for the donor choice mechanism in *S. pombe* differs from donor preference in *S. cerevisiae*, as do other aspects of mating-type switching. The gene conversion of the *MAT* locus in *S. cerevisiae* are not dependent on passage of a replication fork through a nicked *MAT l*ocus as in *S. pombe*; instead, recombination is initiated by the HO endonuclease that creates a double-strand break at its recognition site at *MAT***a** or *MAT*α [reviewed by Haber (2016), Lee and Haber (2015); Fig. 1]. Normally, HO endonuclease is expressed at the end of G1, after passage through “start,” but cells have entered S phase before recombination has been completed (Haber 2016). However, *MAT* switching can be induced both in cells arrested and maintained prior to S phase, when Cdc7 is inactivated (Hicks et al. 2011), or in G2/M-arrested cells (Wang et al. 2004), by expressing the HO endonuclease gene under control of a galactose-inducible promoter.

HO cleavage occurs close to the junction between the Y**a**- or Yα-specific sequences and the Z1 and Z2 homology regions shared between *MAT* and *HML* or *HMR* (Fig. 2). 5′ to 3′ resection of the DSB ends allows Rad51 protein to bind to the single-stranded DNA to promote strand invasion, copying of the donor Y region and termination in the longer shared homology regions W and X. Although *HML* shares more homology with *MAT* than does *HMR*, the 230 bp Z1 region is sufficient to yield nearly 100% recombination (Mehta et al. 2017).

As with *S. pombe*, a cell-type-specific donor selection mechanism can be inferred from the high efficiency of switching to the opposite mating-type in switching-competent cells, in this case, mother cells (Hicks and Herskowitz 1976; Strathern and Herskowitz 1979). The *HML* locus is preferentially selected in **a**-mating cells and the *HMR* locus is chosen in *MAT*α cells. As in *S. pombe*, donor preference is not dependent on the specific mating-type sequences in the donor locus, as the same preference is seen when both donor loci contain Yα or when *HML* contains Y**a** sequences and *HMR* carries Yα (Klar et al. 1982; Rine et al. 1981). Both *HML* and *HMR* are maintained as short, heterochromatic, non-transcribed regions (Fig. 2), but unlike in *S. pombe*, the two loci are located far away from *MAT* and from each other, near the two telomeres of the same chromosome. *MAT* switching, therefore, requires long-range ectopic interactions between loci that are about 200 or 100 kb away from the HO-cut *MAT* locus (Fig. 1). As in *S. pombe*, the silenced donors have deacetylated histones; however, the histone H3K9 methylation pathway does not exist in *S. cerevisiae* and thus does not contribute to gene silencing or donor selection (Fig. 2). Instead, silencing of *HML* and *HMR* relies entirely on the Sir2 histone deacetylase pathway, which plays a less significant role for silencing in *S. pombe*.

Donor preference is controlled from a distance by a cell-type-specific Recombination Enhancer (RE), the ~ 750-bp multipartite *RE* element located 17 kb away from *HML* towards the centromere (Wu and Haber 1996) (Fig. 3). Sequences closer to the donor loci can be deleted with no effect on donor preference (Weiler and Broach 1992; Wu and Haber 1995). When RE is deleted, donor preference shifts from ~ 90% *HML* usage to less than 10%. In *MAT***a** cells, *RE* is bound by the Mcm1 protein (Szeto et al. 1997), the dimeric transcription factor Swi4/Swi6 (Coic et al. 2006) and multiple copies of the forkhead protein Fkh1 (Sun et al. 2002). Mechanistically, RE acts primarily through the several copies of Fkh1 bound to RE (Li et al. 2012). RE can be deleted and replaced by LexA binding sites to which LexA-Fkh1 (or simply the phospho-threonine binding domain of Fkh1) is bound. Fkh1 apparently binds to phospho-threonines produced by a protein kinase on proteins bound at or near the DSB, so that *HML* is effectively tethered ~ 20 kb from the DSB, via RE, while *HMR* remains nearly 100 kb distant (Avsaroglu et al. 2016). One phosphorylated protein, Mph1 (Dummer et al. 2016) has been implicated, but the effect of deleting Mph1 is much less than creating a Fkh1-R80A mutation that abolishes the phospho-threonine-binding of Fkh1 (Li et al. 2012). One protein kinase implicated in this process is casein kinase II (Ck2), but its targets have not been identified and its absence does not have as severe an effect as deleting Fkh1 (Li et al. 2012).

This proposed mechanism of action for *RE*, induced proximity of donor and recipient loci, differs from the facilitation of strand exchange proposed in *S. pombe* for *SRE2* and *SRE3*, but might correspond to a function of *SRE2* and *SRE3* that still remains to be identified. However, a key difference between budding and fission yeast is that in the case of *S. cerevisiae* donor preference remains in full force even when one donor or both donors are unsilenced (Coic et al. 2011). Active RE is a “portable” enhancer of recombination, such that insertion of this short DNA sequence in different chromosomal locations will enhance the use of an ectopic donor even during interchromosomal recombination between unsilenced sequences that are unrelated to *MAT* (Lee et al. 2016; Roy et al. 2018). *SRE2* or *SRE3* are also to some extent ‘portable’ in that they remain active when their position is changed within the mating-type region (Jakociunas et al. 2013), but whether they function at other chromosomal locations to affect other DSB induced recombination events has not been tested.

In *MAT*α cells, the *MAT*α-encoded α2 protein dimerizes with Mcm1 and inactivates *RE* (Szeto et al. 1997); a change in chromatin structure is induced and the bound transcription factors are displaced (Weiss and Simpson 1997). *RE* is thereby inactivated in *MAT*α cells and the default *HMR***a** donor is chosen instead of *HML*α. The preferential use of *HMR* over *HML* in *MAT*α cells (or in *MAT***a** cells deleted for RE) is stronger than one would predict as a function of their relative distances to *MAT* (Avsaroglu et al. 2016), raising the possibility that there could be an unknown *HMR*-adjacent enhancer. An alternative possibility, based on the finding that adjacent to RE is a second Matα2-Mcm1 binding site (Szeto et al. 1997), is that RE could be tethered to another site, preventing it—and thus *HML*—from interacting with *MAT*α. Indeed, artificially tethering a set of LacO repeats between *HML*α and RE to the nuclear periphery by a LacI::FFAT domain does constrain the use of *HML* (Avsaroglu et al. 2014).

In *MAT***a**/*MAT*α diploid cells, Matα2 and the *MAT***a**-encoded **a**1 protein form a heterodimer, which represses haploid-specific genes including the HO gene (Haber 2016). Therefore, switching is repressed in diploids. However, in *S. pombe*, diploids cells can switch mating-type on either chromosome (Egel 1984b; Egel and Eie 1987).

## Cell fate in the absence of donors

Another striking difference between mating-type switching in budding and fission yeasts is the consequence of deleting both donor sequences when a DSB is induced. Induction of HO in an *hml∆ hmr∆* strain results in near-total lethality in *S. cerevisiae* (Klar, et al. 1984), with about 0.2% of cells surviving by nonhomologous end-joining events that mutate the cleavage site (Moore and Haber 1996; Wu et al. 1996). In contrast, *S. pombe* cells deleted for both *mat2-P* and *mat3-M* but competent to undergo switching are viable (Klar and Miglio 1986), though their viability remains dependent on the presence of homologous recombination proteins such as Rad51 or SpRad22 (ScRad52) (Roseaulin et al. 2008). In the absence of the two donors, repair occurs by sister-chromatid recombination with the unbroken *mat1* locus (since the DSB arises by replication through a single-strand nick). These results raise another question about the role of heterochromatin and the *SRE* loci in the repair of the *mat1* DSB: why is sister-chromatid repair not always favored as the means of repair, since there is far more homology on both sides of the DSB than the remarkably short 59-bp H1 region that is used to initiate switching from the donors. These results suggest another role for the chromatin structure of the mating-type cassettes, to prevent sister-chromatid repair. It is interesting to note that, in budding yeast, an HO-induced DSB leads to the recruitment of cohesion around the break, which would promote sister-chromatid recombination (Ström et al. 2004; Ünal et al. 2004). How cohesion recruitment is affected by heterochromatin in the mating-type region in fission yeast would be worth examining. Interestingly, the switch-activating protein Sap1 that binds DNA elements at *mat1* that are required for mating-type switching, is also present at *IR-L* and *IR-R* (Raimondi et al. 2018) and has been proposed to play a role in cohesion (de Lahondes et al. 2003).

## Inhibitory versus stimulatory roles of heterochromatin on recombination

Some forms of recombination are inhibited by heterochromatin. The *HML* and *HMR* loci are protected from cleavage by the HO endonuclease (Strathern et al. 1982; White and Haber 1990), but they are cleaved in *sir* mutants and can then act as gene conversion recipients from an uncleavable *MAT* locus (Haber et al. 1980; Klar et al. 1981). In *S. pombe*, heterochromatin prevents both mitotic and meiotic recombination events. In meiosis, the occurrence of crossovers (CO) is repressed more than a thousand fold in the *mat2-P* - *mat3-M* interval (Egel 1984a, b); this block is released in heterochromatin mutants (Egel et al. 1989; Klar and Bonaduce 1991; Lorentz et al. 1992; Thon and Klar 1992, Thon and Klar 1994) or by deleting the heterochromatin nucleation center *cenH* located between *mat2-P* and *mat3-M* (Grewal and Klar 1997; Klar and Miglio 1986). In vegetative cells, chromosomal integration of transformed DNA is inefficient in the mating-type region and more efficient in heterochromatin mutants (Thon and Klar 1993). These recombination blocks stand in sharp contrast to the efficiency with which the silent donor loci are copied during mating-type switching. Recombination blocks also occur in pericentromeric heterochromatin. In meiosis, heterochromatin prevents COs in centromeric regions in *S. pombe* (Ellermeier et al. 2010, Fowler, et al. 2014), Arabidopsis (Underwood, et al. 2018) and other eukaryotes (reviewed by Nambiar and Smith 2016). In *S. cerevisiae*, meiotic recombination is also repressed near centromeres and in subtelomeric regions (Barton et al. 2008). There is a positive correlation between the location of meiotic hotspots and open chromatin (Berchowitz et al. 2009). The inhibitions have an important biological function, because COs too close to centromeres would interfere with chromosome segregation, or lead to gross genomic rearrangements if the COs occurred between common repeated sequences found on different chromosomes.

The mechanistic bases behind the inhibitions are only partly known. One level of control is through the inhibition of DSB formation. Heterochromatin prevents cutting by many restriction enzymes in addition to the HO endonuclease in the nuclei of mitotically dividing cells, suggesting the existence of steric hindrances (Loo and Rine 1994). Moreover, nucleases that gain close access to DNA might also be inactivated. For instance, in *S. cerevisiae*, tethering the meiotic nuclease Spo11 to various chromosomal locations sometimes results in DSBs but not always (Murakami and Nicolas 2009; Robine et al. 2007). Cohesins can have a determinant role (Nambiar and Smith 2018). In *S. pombe* meiosis, the mitotic cohesin subunit Psc3 is recruited by pericentromeric heterochromatin instead of the meiotic-specific cohesin Rec11 found in chromosome arms. The absence of Rec11 precludes activation of the Rec12(Spo11) pathway, of which Rec11 is an early effector. Thus, pericentromeric heterochromatin prevents DSB formation by Rec12(Spo11) and CO formation (Ellermeier et al. 2010; Fowler et al. 2014; Nambiar and Smith 2018).

One possible explanation for the differential effects of heterochromatin on different forms of recombination would be that nuclease access is prevented but that cells have the necessary chromatin remodelers to allow these same regions to be used as efficient donors (Chai et al. 2005; Hicks et al. 2011; Kent et al. 2007; van Attikum et al. 2004). Heterochromatin might also channel recombination to pathways that do not result in COs, this might be achieved on occasion through the use of distinct mediators of recombination that have the capacity of dictating recombination outcomes (Akamatsu et al. 2007). *Schizosaccharomyces pombe* mating-type switching shows that chromatin structure can affect the choice of recombination accessory factors; for example the poor switching of Swi2 and Swi5 mutants is significantly suppressed by the absence of Swi6 (Jia et al. 2004; Matsuda et al. 2011) indicating heterochromatin normally prohibits the use of alternate facilitators of strand exchange.

## Perspectives

Genetic analyses of mating-type switching have identified a number of elements capable of conditionally imposing strong biases to recombination, some of which should be amenable to detailed in vivo monitoring after synchronized DSB formation as well as in vitro characterization. Might *SRE2* and *SRE3* facilitate strand exchange in vitro and what is their dependency on recombinases and accessory factors? In the future, genetic modifications of histones and biochemical assays using nucleosomal DNA might allow sorting out the effects of various histone modifications and protein complexes, to improve our understanding of how homologous recombination can be enhanced or suppressed.
